# Tiny packages, big potential: bacterial membrane vesicles in vaccinology

**DOI:** 10.1186/s12934-025-02908-5

**Published:** 2025-12-30

**Authors:** Ayşe Varol, Şeyma Aydın, Ahmet Adıgüzel, Selçuk Özdemir

**Affiliations:** 1 Department of Molecular Biology and Genetics, Faculty of Science, Atatürk University, Erzurum, Turkey; 2 Department of Genetics, Faculty of Veterinary Medicine, Atatürk University, Erzurum, Turkey

**Keywords:** Next-generation vaccines, Bacterial membrane vesicles, Outer membrane vesicles, Translational vaccinology, Vaccine platforms

## Abstract

Bacterial membrane vesicles (BMVs) are nanoscale, bilayered proteolipid structures secreted by both Gram-negative and Gram-positive bacteria. Initially considered cellular debris, BMVs are now recognized as evolutionarily conserved entities with critical roles in bacterial communication, immune modulation, virulence factor delivery, and horizontal gene transfer. Their structural and functional resemblance to eukaryotic extracellular vesicles has fueled growing interest in their use as versatile vaccine platforms. Licensed meningococcal OMV vaccines established proof-of-concept for their safety and immunogenicity, and ongoing studies are extending applications to enteric pathogens and viral infections. Recent advances in genetic engineering, glycoengineering, and modular antigen display systems have enabled the design of “plug-and-play” BMVs with reduced reactogenicity and enhanced protective efficacy. In parallel, innovations in bioprocessing and formulation technologies are improving scalability, stability, and delivery, including mucosal routes. This review highlights the immunological properties, translational potential, and key challenges of BMV-based vaccines, with an emphasis on strategies to optimize safety, antigen specificity, and manufacturing for next-generation vaccine development.

## Introduction

Having existed for billions of years, bacteria are among the most ancient and diverse microorganisms on Earth. Prokaryotic cells, typically ranging from 0.5 to 5 μm in size, are exceptionally adaptable to diverse environmental conditions. Recent studies have demonstrated that bacteria, eukaryotes, and archaea all secrete nanometer-scale membrane vesicles into the extracellular environment. These evolutionarily conserved structures range from 20 to 400 nm and are spherical, bilayered proteolipid vesicles carrying defined subsets of proteins, lipids, nucleic acids, and metabolites [1].

Bacterial membrane vesicles (BMVs) are secreted by both Gram-positive and Gram-negative bacteria and participate in numerous biological processes, including intercellular communication, pathogenicity, biofilm formation, horizontal gene transfer, protein transport, and stress responses [2]. Gram-negative bacteria possess a multilayered envelope consisting of an outer membrane (OM) enriched with lipopolysaccharides (LPS), a thin periplasmic peptidoglycan (PG) layer, and an inner membrane (IM). In contrast, Gram-positive bacteria contain a thick PG layer surrounding the cytoplasmic membrane. Over the years, the literature has identified several BMV subtypes based on their biogenesis or method of production. Gram-negative bacteria predominantly produce outer membrane vesicles (OMVs) derived from the OM, whereas Gram-positive bacteria generate vesicles that bud from the cytoplasmic membrane and traverse the thick PG layer, commonly referred to as membrane vesicles (MVs). Additional vesicle types have been described, including double-membrane vesicles (DMVs) or bacterial biomimetic vesicles (BBVs) formed through physical or chemical disruption of bacterial cells, and protoplast-derived nanovesicles (PDNVs) or cytoplasmic membrane vesicles (CMVs) that originate from the inner membrane following removal of the OM. Moreover, minicells, large anucleate vesicle-like particles, arise from aberrant bacterial division [3].

BMVs were first observed in the 1960s through electron microscopy and were initially regarded as cellular waste because their biological functions were unknown [4]. In the 1970s and 1980s, vesicular structures were shown to contain outer membrane proteins, enzymes, and LPS, yet their purpose remained unclear [5]. Breakthroughs in molecular biology during the 1990s revealed that BMVs play essential roles in virulence factor secretion, antibiotic resistance, genetic material transfer, and intercellular communication [6, 7]. These discoveries renewed scientific interest in the 2000s, positioning BMVs as important biological entities with potential applications in biotechnology, vaccine development, and drug delivery [8, 9]. Today, membrane vesicles from both Gram-negative and Gram-positive bacteria are recognized as evolutionarily conserved, tightly regulated nanosystems with diverse functional roles.

Contrary to early assumptions, BMVs are now understood to be key mediators of bacterial communication, immune modulation, virulence factor delivery, and horizontal gene transfer insights made possible through advances in molecular biology, proteomics, and high-resolution imaging [10]. Their nanoscale size, bilayered composition, and ability to carry biomolecules closely resemble the properties of eukaryotic extracellular vesicles [11], making BMVs highly attractive candidates for translational applications in drug delivery, diagnostics, and vaccine design.

This review focuses on the emerging role of BMVs in vaccine development. We first summarize their immunological properties, including their intrinsic adjuvant activity, natural display of pathogen-associated molecular patterns (PAMPs), and capacity to induce coordinated innate and adaptive immune responses. We then examine engineering strategies that enhance the safety and efficacy of BMVs, such as genetic detoxification approaches to reduce endotoxin content, modular surface display of heterologous antigens, and the development of synthetic bacterial biomimetic vesicles. Finally, we highlight recent progress in preclinical and clinical studies evaluating BMVs as next-generation platforms for bacterial, and viral vaccines, outlining both the opportunities and challenges associated with their clinical translation.

Although several prior reviews have examined BMVs, none has integrated mechanistic immunology, antigen-engineering technologies, biomanufacturing innovations, and mucosal delivery strategies within a unified and up-to-date framework. By connecting these rapidly evolving domains, this review provides the first comprehensive synthesis of how modern genetic detoxification, plug-and-play antigen-display systems, and next-generation generalized modules for membrane antigens (GMMA) technologies collectively redefine BMVs as modular, scalable, and clinically feasible vaccine platforms.

## Biogenesis of the BMVs

### Extracellular vesicles of gram-negative bacteria: OMVs

In the 1960s, electron microscopy investigations of bacterial architecture led to the discovery of MVs, also known as OMVs, which are produced from Gram-negative bacteria. It was first demonstrated that the OM of Gram-negative bacteria is the source of these extracellular vesicles, which have a diameter ranging from 20 to 200 nm. Two main routes for MV biogenesis in Gram-negative bacteria are currently understood to exist: (i) type B MVs, which are vesicles created by blebbing of the outer membrane, and (ii) type E MVs, which are produced by folding and spontaneously reorganizing fragmented membrane remnants and are typically linked to explosive cell lysis [12].

Explosive cell lysis, which is frequently brought on by genotoxic stress, is characterized by membrane lysis and vesicle formation. Prophage-derived endolysins are then activated, which destroys the peptidoglycan layer. Lytic bacteriophages can either directly cause MV synthesis by producing MV vesicles or interact with MV in a similar way, resulting in explosive lysis.

These many biogenesis processes influence the biological roles of MVs by causing them to arise with a variety of structures and compositions. For instance, type B OMVs typically do not contain cytosolic components like DNA, RNA, or ATP, whereas OMVs originating from outer membrane blebs frequently carry hydrophobic substances, misfolded proteins, or peptidoglycan fragments. On the other hand, cytoplasmic CMVs from Gram-positive bacteria or type E MVs from Gram-negative bacteria are more likely to include such cytosolic materials [13].

### Extracellular vesicles of gram-positive bacteria

MVs were thought to be exclusive to Gram-negative bacteria for a long time since it was believed that the thick peptidoglycan layer of Gram-positive bacteria inhibited the release of membrane-derived vesicles. However, current research employing proteomic analysis and transmission electron microscopy has directly demonstrated that Gram-positive bacteria are also capable of actively producing MVs (also known as cytoplasmic membrane vesicles, or GVs or CMVs).

Vesicle biogenesis occurs in Gram-positive bacteria once the strong peptidoglycan layer locally weakens and the cytoplasmic membrane budding outward. Vesicle release is facilitated by the involvement of autolysins, hydrolases, and peptidoglycan-remodeling enzymes.

Several biological processes are carried out by the resultant vesicles. These include crucial functions such genetic material transfer, stress response and environmental adaptation, virulence factor trafficking, and interaction with the host immune system [14, 15].

## The molecular components of the BMVs

**Fig. 1:**
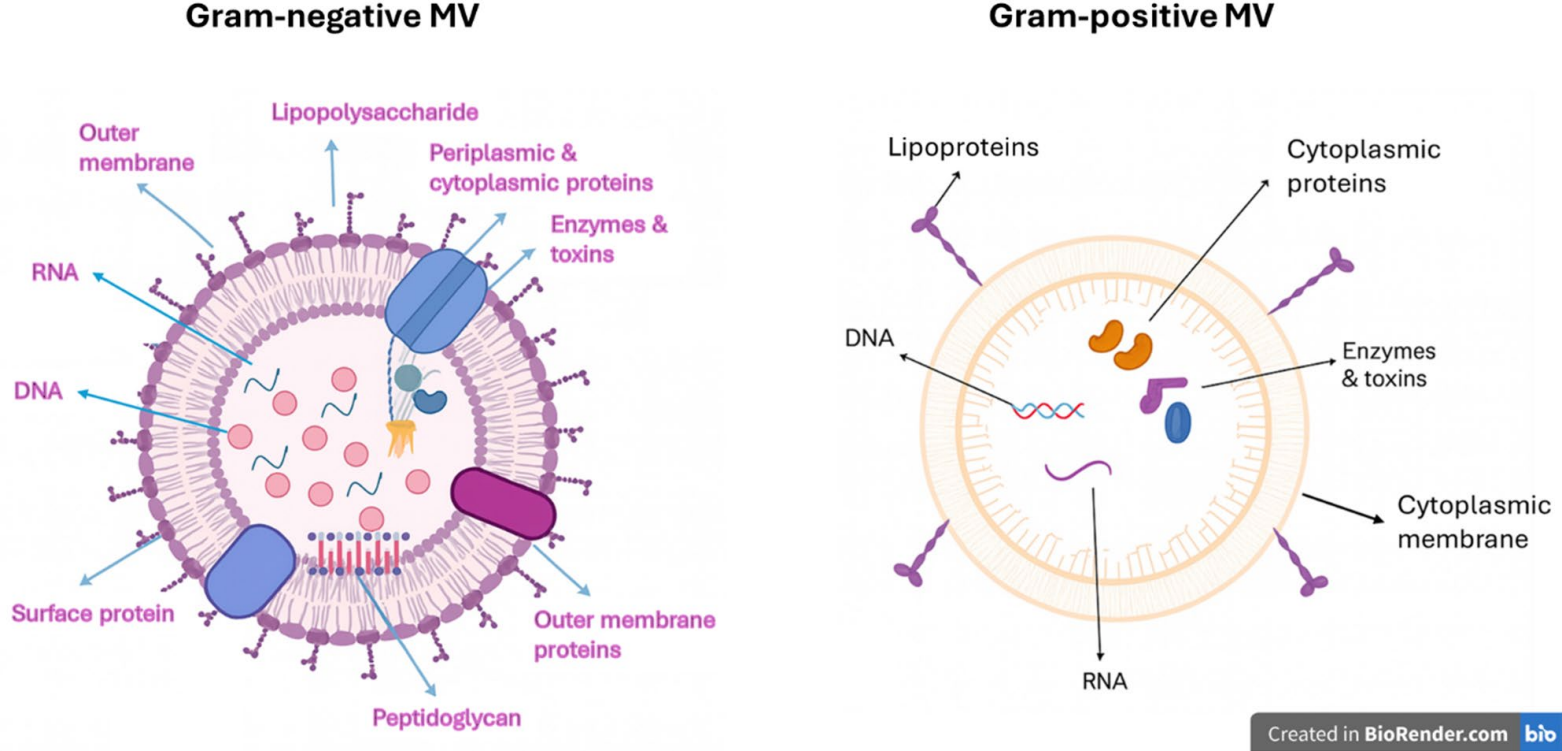
MVs from both Gram-positive and Gram-negative bacteria: their structure. In addition to carrying peptidoglycan, enzymes, toxins, cytoplasmic proteins, and nucleic acids, gram-negative MVs are covered with LPS and outer membrane proteins. Lipoproteins, enzymes, toxins, cytoplasmic proteins, and nucleic acids are all present in gram-positive MVs, which are derived from the cytoplasmic membrane

Proteins, lipids, nucleic acids, LPS, and other genetic materials are all found in BMVs. Current understanding of these vesicular components is compiled in the ensuing subsections (Fig. 1).

### Lipids

Research on both Gram-positive and Gram-negative bacteria has shown that the lipid makeup of their membrane vesicles is different from that of their host membranes. OMVs are abundant in phosphatidylethanolamine, LPS, and phospholipids found in the outer membrane of Gram-negative bacteria. Modified lipid A species, which are involved in immune recognition, are also commonly found in them. In contrast, phosphatidylglycerol, cardiolipin, and other lipids linked to membranes are present in vesicles that are produced from Gram-positive bacteria [16].

The first findings on lipid composition were obtained by Horstman and Kuehn using thin-layer chromatography (TLC), demonstrating that phosphatidylglycerol, glycerophospholipids, phosphatidylethanolamine, and cardiolipin are the main lipid components in enterotoxigenic *Escherichia coli* extracellular vesicles. Later, Chowdhury and Jagannadham, using mass spectrometry-based lipidomic analysis, demonstrated that even-numbered carbon chain phosphatidylglycerol and phosphatidylethanolamine were the predominant lipids in the membrane bilayer of *Pseudomonas syringae* extracellular vesicles [17, 18].

### Proteins

OMVs from Gram-negative bacteria have been shown to contain numerous outer membrane proteins (OMPs such as OmpA, OmpC, and OmpF), periplasmic proteins (e.g., AcrA and alkaline phosphatase), misfolded proteins, and virulence factors involved in adhesion and invasion in host tissues. These findings were demonstrated in early studies on membrane vesicles using biochemical analyses such as protein staining, polyacrylamide gel electrophoresis (PAGE), and Western blotting [19]. Subsequent studies have conducted comprehensive proteomic analyses of OMVs from both pathogenic and commensal bacteria, revealing novel functional proteins with potential biomedical applications [20].

### Genetic burden and nucleic acids

Analyses conducted following DNase treatment verified the existence of DNA in OMVs, indicating that luminal DNA is resistant to this treatment. DNA, RNA, plasmids, phage DNA, and chromosomal DNA can all be carried by bacterial OMVs in the luminal region and attached to the surface. In fact, OMVs from *Haemophilus influenzae (H. influenzae)*,* Pseudomonas aeruginosa (P. aeruginosa*), *Neisseria gonorrhoeae (N. gonorrhoeae)*, and *Escherichia coli (E. coli)* have been found to contain different types of luminal DNA. It has also been demonstrated that OMVs from the commensal bacterium Enterobacter cloacae contain genomic DNA.

It is believed that by taking part in horizontal gene transfer activities, the genomic DNA found in OMVs may aid in the evolution of microbes. It is hypothesized that genomic DNA fragments promote horizontal gene transfer and the development of antibiotic resistance by facilitating genetic material interchange across bacteria. Additionally, transposable DNA elements are disseminated more easily by OMVs carrying genomic DNA, which helps bacteria adapt to stressful environments [21–23].

It has also been revealed in recent years that OMVs contain tiny non-coding RNAs (sRNAs), which are between 50 and 400 nucleotides long. Ghosal and associates showed that OMVs produced by *E. coli* MG1655 contained sRNAs with RNA fragments that ranged in size from 15 to 40 nucleotides. Similarly, OMVs from *P. aeruginosa*,* uropathogenic* E. *coli* strain 536, and Vibrio cholerae have also been found to contain sRNAs. Moreover, it has been demonstrated that sRNA-loaded OMVs are secreted by the periodontal pathogens *Treponema denticola*,* Porphyromonas gingivalis*, and *Aggregatibacter actinomycetemcomitans* [7, 24].

Nevertheless, it was discovered that the sRNAs contained in OMVs produced by *Salmonella typhimurium* were resistant to the ribonuclease (RNase) enzymes. All of these findings imply that bacteria use OMVs to transport functional sRNAs in order to shield them from RNase breakdown and to use them in host or bacterial biological activities [25].

### Lipopolysaccharides (LPS)

One of the most characteristic elements of membrane vesicles is LPS, which is found in the outer membrane of Gram-negative bacteria. Lipid A, core oligosaccharide, and O-antigen chain are the three primary sections that make up the LPS structure. This structure is crucial in determining the vesicles’ immunological characteristics. According to reports, the initial lipid A structures were elucidated in members of the Enterobacteria [26, 27].

The toxic effect is caused by lipid A, which also strongly stimulates the host immunological response through TLR4. Ensuring structural integrity is the role of the core oligosaccharide structure. Complement resistance and antigenic diversity are significantly influenced by the O-antigen [28].

Despite the fact that OMVs’ lipid content is largely comparable to that of the OM they come from, there are some significant species-specific variations. According to research on *Escherichia coli*, phosphatidylethanolamine (PE) is the main phospholipid found in OMVs, and they also have greater concentrations of phosphatidylglycerol (PG) and lyso-PE than the cytoplasmic membrane. The phospholipid contents of OM and OMVs for *Helicobacter pylori* are essentially the same, according to analyses conducted with different techniques [29]. Various Gram-negative bacteria have various OMV lipid patterns. While PG and PE were shown to be the most prevalent lipids *in Neisseria meningitidis* OMVs, *Pseudomonas aeruginosa* OMVs were found to have higher quantities of PG and stearic acid than OM, which was linked to increased membrane stiffness. High levels of lyso-PE were found in both OM and OMV in comparison to the inner membrane, whereas PE and, to a lesser extent, cardiolipin were shown to be the main lipid components in *H. pylori* OMVs. This lipid type makes up around 10% of the total membrane lipids in *H. pylori*, one of the few bacteria that contains cholesterol, drawing attention to the bacteria [14].

The various bacteria have different phospholipid distributions. Phospholipids are typically located in the inner leaflet of the OM in *E. Coli* and other Enterobacteriaceae, although they can also be found in the outer leaflet in certain species. LPS, on the other hand, are found only on the outside of OM. Interestingly, it has been observed that OM contains both A- and B-band LPS, but *P. aeruginosa* OMVs solely contain B-band LPS [30].

## Intrinsic adjuvanticity of BMVs

A critical interpretation of the comparative studies suggests that the enhanced immunogenicity observed when OMVs are co-administered with heterologous antigens does not arise from a single mechanistic feature, but rather from the synergistic integration of multiple immunological cues within the same vesicular platform. Unlike alum or CpG, which stimulate isolated pathways (NLRP3 inflammasome for alum, TLR9 for CpG), OMVs deliver a concurrent combination of microbial-associated molecular patterns (MAMPs), including LPS, lipoproteins, and bacterial DNA, each capable of activating distinct pattern recognition receptors (PRRs) [31–33]. This multi-ligand activation generates a more physiologically relevant immune profile, mimicking the complexity of natural bacterial encounters and promoting a more durable and polyfunctional adaptive response.

Another important consideration is the spatial and structural arrangement of antigens on OMVs. OMVs present antigens in a membrane-integrated form that is topologically similar to their native bacterial state. This organization may facilitate more effective B-cell receptor engagement compared to soluble antigens delivered with alum or CpG, which lack a defined structural context. The particulate nature of OMVs (typically 20–300 nm) also positions them within the optimal size range for lymphatic drainage and passive accumulation in lymph nodes, where they interact efficiently with resident dendritic cells [31, 34]. This lymph node–targeting property gives OMVs an innate advantage in initiating early immune priming.

Moreover, OMVs appear to enhance cross-presentation, a process required for strong CD8⁺ T-cell activation [35, 36]. Based on existing data, the endosomal escape or membrane fusion capability of OMVs enables cytosolic access of antigenic cargo [37]. This is a critical distinction because CpG, despite its strong TLR9 activation profile, remains confined to endosomal compartments and therefore only weakly supports cross-presentation [38, 39]. In contrast, OMVs inherently provide both antigen and adjuvant signals in a form that is efficiently routed into major histocompatibility complex class I (MHC-I) and MHC-II pathways simultaneously [40]. This dual loading likely explains the superior cellular immunity observed in several studies.

In addition to these molecular mechanisms, OMVs may contribute to functional tuning of dendritic cells, influencing their cytokine profiles and polarization outcomes. Alum typically skews immune responses toward Th2 dominance, whereas CpG promotes Th1-biased responses [41]. OMVs, however, induce a more balanced or even mixed Th1/Th17 profile, which is advantageous for clearing intracellular pathogens and for generating robust memory responses [42]. This broader activation profile might be a key reason why OMV-adjuvanted formulations outperform single-pathway stimulants.

Taken together, the enhanced efficacy of OMV-based adjuvants derives from their ability to act as self-contained, multi-dimensional immunostimulatory units. They integrate antigen presentation, innate immune activation, intracellular delivery, and lymphatic targeting within one natural nanostructure. This holistic mode of action, rather than a single adjuvant effect, likely underpins their superior performance compared to heterologous antigens delivered with alum or CpG.

## Licensed BMV vaccines: lessons from meningococcal OMVs

One of the most established applications of BMVs as vaccines comes from their use in meningococcal disease control. OMV-based vaccines were originally developed to combat outbreaks of *Neisseria meningitidis* serogroup B [43]. In these formulations, native OMVs were detoxified and enriched for immunodominant outer membrane antigens, including porins and lipooligosaccharides. Several OMV vaccines, most notably those deployed in New Zealand and Cuba and later adapted for European and North American markets, demonstrated that BMVs can elicit robust serum bactericidal antibodies in humans [44, 45]. These early successes provided the first real-world evidence that vesicle-based vaccines can be manufactured at scale, are safe, and offer meaningful protection during epidemics.

A landmark example is the MeNZB vaccine implemented in New Zealand [46]. Designed specifically to control a prolonged serogroup B epidemic that began in 1991, MeNZB was administered between 2004 and 2006 to individuals under 20 years of age, with continued routine immunization of infants and preschoolers until 2008. The program concluded in 2011 after targeting individuals at high medical risk. Evaluation of the campaign estimated a 77% effectiveness after three doses, with a mean follow-up of 3.2 years [47]. These findings highlight not only the public-health impact of OMV vaccines but also their favorable safety and immunogenicity profiles when deployed in large populations under real epidemic conditions.

However, these favorable outcomes of the MeNZB vaccine cannot be attributed solely to the OMV component. In addition to the intrinsic immunostimulatory properties of meningococcal OMVs, the MeNZB formulation included aluminum salts as an adjuvant to ensure sufficient immunogenicity [48]. Aluminum hydroxide played a crucial role by adsorbing OMV antigens, creating a depot at the injection site, and enhancing antigen uptake by dendritic cells, ultimately promoting T-helper cell activation and robust antibody production. This adjuvant effect was particularly important in infants and young children, who typically generate weaker immune responses to OMV antigens alone. Clinical and post-licensure data from the New Zealand mass vaccination campaign further demonstrated that the aluminum-adjuvanted OMV formulation elicited high serum bactericidal antibody titers and conferred substantial population-level protection while maintaining an acceptable safety profile [49]. Together, these findings emphasize that the success of MeNZB resulted from the synergistic combination of OMV-based antigenic stimulation and aluminum-adjuvant–mediated immune enhancement, underscoring the importance of integrated vaccine formulation strategies in achieving optimal protective immunity.

Several adjuvant systems have been evaluated as partners for OMV-based vaccines to further enhance or modulate their immunogenicity. Beyond alum, numerous experimental studies have explored additional adjuvants, including MPLA (a detoxified TLR4 agonist), CpG oligodeoxynucleotides (TLR9 agonists) [50], MF59 [51], and chitosan for mucosal delivery [52]. These examples indicate that OMVs can be flexibly combined with a wide range of adjuvant systems depending on the desired immune profile, target population, and route of administration.

OMVs present antigens in a nanoscale vesicular format (typically 20–300 nm), which facilitates rapid lymphatic drainage and direct access to resident dendritic cells. Furthermore, the vesicle membrane clusters antigens in a quasi-native orientation, enabling stronger B-cell receptor crosslinking than soluble antigens. The presence of multiple microbial ligands, such as LPS (TLR4), triacylated lipoproteins (TLR2), and bacterial DNA (TLR9), activates several innate pathways simultaneously, generating a cytokine environment that favors both Th1 and Th17 responses. Mechanistically, OMVs also promote cross-presentation by enabling endosomal escape or membrane fusion, allowing antigen entry into the cytosolic MHC-I pathway, a feature that alum and CpG do not efficiently support.

## Expanding beyond meningococcus: GMMA platforms for enteric pathogens

Beyond meningococcus, BMVs have been advanced as platforms against enteric pathogens such as *Shigella* and *Salmonella* (Table 1). Here, GMMAs, essentially engineered OMVs optimized for vaccine use, are employed. These vesicles are produced by genetically modifying bacterial strains to enhance vesicle release while detoxifying endotoxic components of LPS. For example, deletion of the *msbB* or *htrB* genes reduces the acylation state of lipid A, lowering reactogenicity without compromising immunogenicity [53]. Clinical trials with Shigella GMMA vaccines have shown strong induction of serum bactericidal antibodies in humans, with favorable safety and tolerability profiles [54]. Specifically, the study reported that antibodies with bactericidal activity were detected in vaccinated individuals (but not in placebo recipients), that titers increased with higher doses of O-antigen/protein, and that these functional antibodies remained elevated for up to six months post-vaccination. In addition, the Phase 2a trial conducted in a Shigellosis-endemic region confirmed the vaccine’s favorable safety and tolerability profile. Reactogenicity was acceptable, with mostly mild local and systemic reactions. No serious adverse events were reported. Functional antibody responses, including serum bactericidal activity, were again observed and correlated with anti-LPS IgG levels [55]. This represents one of the most advanced non-meningococcal applications of BMV-based vaccines currently in human testing.

**Table 1:** Clinical trials and licensed applications of BMV-based vaccines referenced in this review

Vaccine / platform	Target pathogen	BMV Type	Status	Key findings (from review)	Clinical trial identifier / notes
MeNZB	*Neisseria meningitidis* serogroup B	Native OMV (detoxified) + Alum	Licensed (New Zealand)	77% effectiveness after 3 doses; strong SBA titers; excellent safety in mass vaccination [46,47]	*No NCT (implemented as a national immunization program)*
rMenB + OMV NZ (MeNZB-derived)	*Neisseria meningitidis serogroup B*	Recombinant MenB antigens + OMV NZ	Phase 3	Booster immunogenicity and safety study; evaluates SBA titers and tolerability	*NCT06995430*
VA-MENGOC-BC (Cuba)	*N. meningitidis* B + C	Native OMV	Licensed (Cuba, Latin America)	Robust SBA responses; widespread field use; outbreak control [45]	*No NCT (licensed public-health vaccine)*
1790GAHB (Shigella sonnei GMMA)	*Shigella sonnei*	GMMA (ΔmsbB detoxified)	Phase 1 Completed	Strong anti-LPS IgG; dose-dependent SBA; favorable safety profile [54]	NCT02017899
altSonflex1-2-3 (multi-serotype Shigella GMMA)	*Shigella flexneri* + *S. sonnei*	Multi-serotype GMMA	Phase 2a Completed	Functional bactericidal antibodies; mild reactogenicity; strong correlation with anti-LPS IgG [55]	NCT03089879
Bivalent Shigella GMMA (*S. sonnei + S. flexneri 2a*)	*Shigella spp.*	O-antigen–enriched GMMA	Phase 1/2	Safe; functional antibodies; supports multivalent GMMA design	NCT04437019
Salmonella Typhimurium GMMA	*Salmonella Typhimurium*	Detoxified GMMA	Phase 1 (ongoing)	Early strong IgG response; promising tolerability	NCT05449098
Salmonella Paratyphi A GMMA	*S. Paratyphi A*	Detoxified GMMA	Phase 1 Completed	High O-antigen IgG titers; good tolerability	NCT05624412

## Antigen engineering and modular vaccine design

Advanced modular antigen-display systems have significantly expanded the versatility of OMV-based vaccines, enabling a true “plug-and-play” design philosophy. Among these, the SpyTag/SpyCatcher bioconjugation system stands out as one of the most technically powerful and flexible tools. SpyTag, a 13-aa peptide, spontaneously and irreversibly forms an isopeptide bond with its protein partner SpyCatcher under physiological conditions. When SpyCatcher is displayed on the surface of engineered OMVs, typically via fusion to outer membrane proteins (e.g., OmpA, ClyA, or autotransporters), any antigen expressed separately as SpyTag-fused protein can be covalently attached in a site-specific, orientation-controlled, and highly efficient manner. This enables OMVs to carry complex eukaryotic antigens that cannot be produced in bacterial compartments, such as the correctly folded and glycosylated SARS-CoV-2 RBD. The resulting OMV-RBD constructs have shown strong mucosal and systemic immunity following both intranasal and intramuscular administration, together with favorable safety profiles [56].

**Fig. 2:**
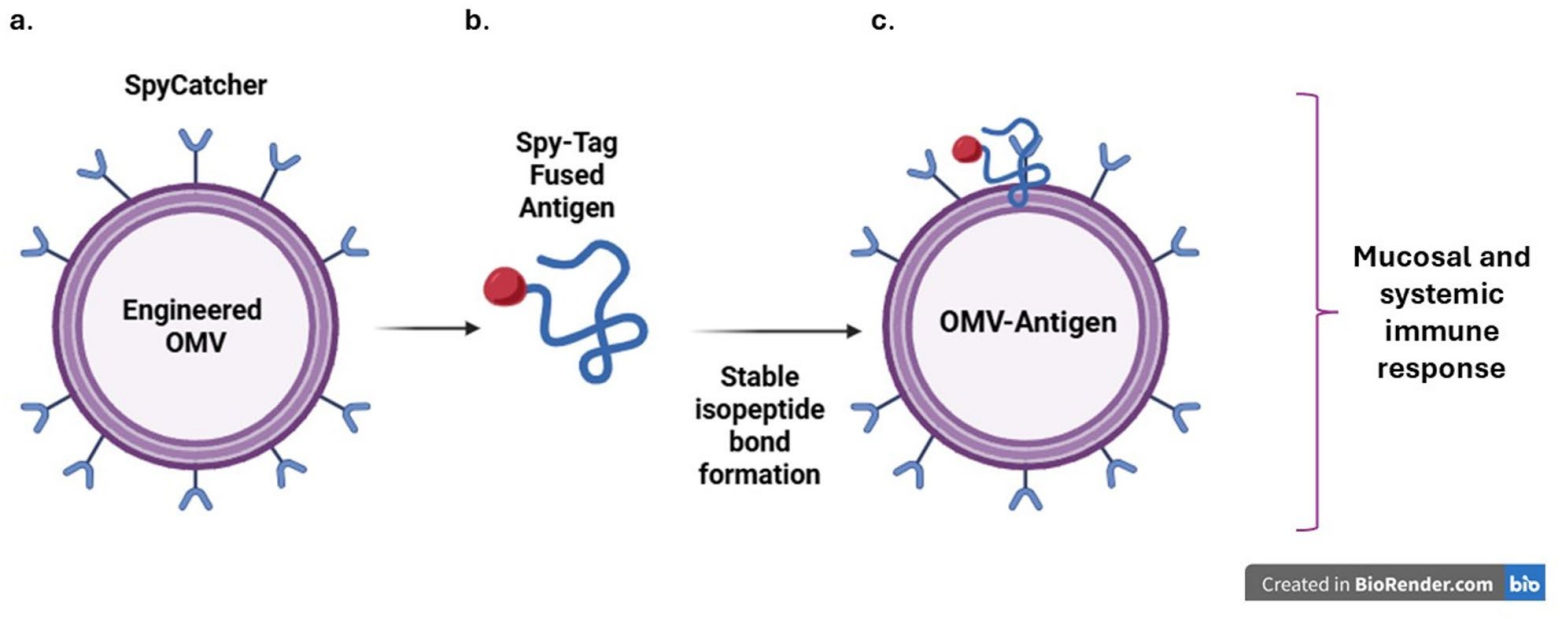
SpyTag/SpyCatcher-based modular antigen display on engineered OMVs. **a** Engineered OMVs expressing SpyCatcher on their outer membrane surface. **b** SpyTag-fused antigen produced separately (typically in a eukaryotic system) approaches the OMV surface. **c** Covalent and irreversible isopeptide bond formation between SpyTag and SpyCatcher results in a stable OMV–antigen complex, enabling high-density, orientation-controlled antigen display. This modular platform supports the induction of both mucosal and systemic immune responses

From a mechanistic perspective, SpyTag/SpyCatcher-engineered OMVs offer several important advantages that enhance their utility as modular vaccine platforms. The system enables covalent and irreversible attachment of antigens, preventing antigen loss or shedding after formulation (Fig. 2). It also allows precise control over antigen density and spatial organization on the OMV surface, an important factor in optimizing B-cell receptor engagement. Because the antigen is produced separately, typically in a eukaryotic expression system, the approach is fully compatible with large, multi-domain, or glycosylated proteins that cannot be efficiently synthesized within bacterial hosts. Moreover, the platform provides remarkable batch-to-batch modularity, allowing rapid antigen exchange without redesigning or reconstructing the OMV scaffold itself. Collectively, these mechanistic features make the SpyTag/SpyCatcher system particularly well suited for rapid-response vaccine development, such as during emerging infectious disease outbreaks, and for the streamlined generation of multivalent vaccine candidates. These mechanistic advantages have been validated across several recent OMV–SpyTag/SpyCatcher vaccine studies [32, 57].

Complementing this approach, other plug-and-play systems have also been established. Engineered *E. coli* OMVs have been designed to simultaneously display multiple antigens, including fluorescent model antigens (mCherry, GFP) and SARS-CoV-2 RBD, using fusion-based strategies that allow co-display of diverse proteins on one vesicle, resulting in stronger humoral responses compared to single-antigen constructs [58]. The ClyA fusion system, which anchors antigens to the OMV membrane by exploiting the pore-forming cytotoxin ClyA, has enabled the display of influenza M2e antigen on probiotic *E. coli* OMVs; vaccinated mice exhibited complete protection from lethal influenza challenge [59]. Likewise, a dual-pathogen OMV vaccine was generated by fusing influenza hemagglutinin with the MERS-CoV RBD, demonstrating robust neutralizing antibody production and survival against heterologous challenges [60].

The hemoglobin protease (Hbp) autotransporter platform represents another high-capacity display system, enabling dense, multivalent presentation of large or repeated antigen domains. OMVs engineered with pneumococcal antigens displayed via Hbp provided significant protection against nasal colonization following intranasal vaccination, a strong example of OMVs’ suitability for mucosal immunization [61] .

Taken together, these modular bioconjugation and display strategies illustrate the emerging landscape of highly customizable OMV vaccine platforms. Each system, SpyTag/SpyCatcher, ClyA, autotransporters, or multi-antigen fusion constructs, provides a unique route for presenting structurally diverse antigens, enabling rapid, flexible, and immunologically potent vaccine development across pathogens, and mucosal diseases.

## Manufacturing and downstream processing innovations

From a manufacturing standpoint, BMVs, particularly GMMA, offer both strengths and challenges. Their production leverages well-established bacterial fermentation processes that are easily scalable using standard bioreactors, often with genetically engineered strains (e.g., deletions in *tolR*, *galU*, or lipid A-modifying genes) to enhance vesicle yield and reduce toxicity. In a GMP-scale process for *Shigella sonnei* GMMA, researchers reported robust yields reaching approximately 100 mg of GMMA protein per liter of fermentation [55]. Moreover, upgrading from batch to continuous bioprocessing in *Neisseria meningitidis* has been shown to increase volumetric productivity nearly ninefold, while maintaining vesicle quality over prolonged operation [61].

However, BMVs present challenges in downstream processing due to their particulate nature and inherent heterogeneity in size and composition. Traditional purification approaches, like ultracentrifugation and size-exclusion chromatography, can ensure high purity but are labor-intensive, low-yielding, and difficult to scale industrially [61, 62]. To address these limitations, more scalable and efficient methods are being explored, including tangential-flow filtration (TFF), membrane chromatography, and multimodal chromatography [62]. For example, innovative gradient filtration techniques using graded pore-size membranes have demonstrated higher yields and preserved functional vesicle properties, nearly doubling recovery compared to ultracentrifugation, making them promising for large-scale operations [63].

## Safety, reactogenicity, and tolerability of BMV-based vaccines

### Genetic detoxification strategies to reduce reactogenicity

Safety optimization has become a central priority in the development of BMV-based vaccines due to the intrinsic reactogenicity of LPS, particularly its lipid A moiety. To mitigate this, modern OMV and GMMA platforms employ rational genetic detoxification strategies. Deletion of lipid A acyltransferases such as htrB (lpxL) and msbB generates penta-acylated lipid A variants with substantially reduced TLR4-driven inflammatory signaling compared to the highly stimulatory hexa-acylated wild-type structure. Additional attenuation is achieved using deep-rough mutants, which lack extended O-antigen polysaccharide chains, thereby decreasing endotoxicity and enhancing the exposure of outer membrane antigens [64, 65]. Collectively, these molecular engineering strategies form the foundation of next-generation, low-reactogenicity BMV vaccine platforms.

### Preclinical and clinical evidence supporting safety and tolerability

Robust preclinical toxicology data corroborate the favorable tolerability of detoxified BMVs. OMVs derived from S. typhimurium ΔmsbB or ΔpagP mutants exhibit markedly reduced pyrogenicity, minimal increases in body temperature, and lower IL-6/IL-1β secretion compared to wild-type OMVs. Dose-escalation and repeat-dose studies consistently report no morbidity, mortality, or organ toxicity, with normal histopathology in the liver, spleen, and draining lymph nodes [66].

Similarly, Shigella GMMA vaccines engineered with ΔtolR and ΔhtrB mutations demonstrate benign safety profiles across multiple models. Human PBMC assays show diminished IL-6 responses, while rabbit pyrogenicity, repeated-dose intramuscular, intradermal, and intranasal studies confirm minimal systemic inflammation, stable clinical chemistry, and no delayed toxicity [55].

Human studies further validate the safety of intranasally administered meningococcal OMVs. In a clinical trial evaluating a MenB OMV vaccine delivered as nasal drops or spray, subjects developed strong mucosal IgA responses with only mild and transient nasal irritation, and no serious adverse events. Serum IgG and bactericidal antibodies persisted for months, demonstrating that OMVs are both immunogenic and well tolerated in humans [67].

### Safety of mucosal (intranasal and oral) delivery routes

Multiple animal studies corroborate the safety of mucosal delivery. Intranasal OMV administration in mice induces high levels of IgA in lung lavage fluid and robust serum bactericidal antibody responses without excessive inflammatory cytokine release [68]. Rabbits immunized intranasally with *Neisseria meningitidis* OMVs similarly develop strong mucosal and systemic immunity without respiratory or systemic toxicity [69]. OMVs derived from *Bordetella pertussis* have also been shown to reduce nasal colonization in mice with no adverse effects, highlighting the platform’s capacity to safely stimulate respiratory mucosal immunity [70].

Oral strategies are more challenging due to degradation in the gastrointestinal tract, but innovative approaches have been explored. For example, *Shigella flexneri* OMVs encapsulated in biodegradable nanoparticles (poly-anhydride formulations) were tested in mice. Intranasal and conjunctival administration of these formulations provided strong protection, whereas free OMVs were less effective. Importantly, encapsulated OMVs triggered protective immune responses without causing significant systemic inflammation [71].

OMVs are particularly suitable for intranasal and oral delivery because their nanoscale size and membrane composition allow them to efficiently cross mucosal barriers and be taken up by M cells and dendritic cells within Peyer’s patches and nasal-associated lymphoid tissue (NALT) structures. The simultaneous engagement of TLR2, TLR4, and NOD receptors at these sites creates a strong local immunoregulatory microenvironment rich in TGF-β and BAFF/APRIL, which are known drivers of IgA class-switch recombination [72–74]. Moreover, OMVs themselves engage innate receptors and deliver PAMPs via M-cells/DC-mediated uptake [72]. Therefore, the robust IgA response observed after mucosal OMV immunization likely reflects genuine antigen-specific IgA produced via classical sequential class switching (IgM→IgA) in germinal centers. In our view, OMVs act not only as antigen carriers but also as potent local inducers of the cellular signals required for IgA diversification and affinity maturation, explaining their superior performance compared to soluble antigens or alum-adjuvanted formulations delivered mucosally.

### Encapsulation technologies enhancing oral and mucosal tolerability

Because oral vaccines must withstand degradation in the gastrointestinal tract, innovative formulation strategies have been developed to improve OMV stability and tolerability. Biodegradable poly(anhydride) nanoparticle, encapsulated OMVs (NP-OMVs) significantly enhance mucosal protection while reducing systemic inflammation. In mice, nasal delivery of NP-OMVs increases survival from 40% to 100% at lower doses, while oral administration of NP-OMVs provides full protection, effects not achieved by free OMVs [75].

Recent work further supports the feasibility of oral OMV vaccines. A tetravalent oral Shigella OMV vaccine induced strong systemic and mucosal antibody responses and protected mice from lethal challenge without triggering systemic toxicity [76]. These outcomes highlight the ability of encapsulation technologies to protect OMVs from gastrointestinal degradation while preventing excessive systemic immune activation.

## Engineering next-generation vesicle-based vaccines

Next-generation BMV vaccines mark a significant advancement over the early meningococcal OMV formulations, addressing prior limitations related to reactogenicity, antigenic breadth, and manufacturing consistency. A major innovation is the genetic detoxification of lipid A through targeted mutagenesis of acyltransferases such as lpxL, lpxM, and msbB, producing penta-acylated lipid A structures with markedly reduced endotoxicity while preserving intrinsic immunostimulatory activity. This refinement increases tolerability and expands the suitability of BMVs for diverse populations.

Parallel developments in synthetic biology have enabled the precise incorporation or surface display of heterologous antigens, converting BMVs into highly modular platforms. OMVs engineered with lipoprotein transport machinery to present *Streptococcus suis* antigens elicit robust immunity in vivo [52], while similar systems have been deployed for viral and bacterial antigens from multiple pathogens. These strategies highlight the broad utility of BMVs as carriers of structurally diverse epitopes.

Among the most promising innovations are glycoengineered OMVs (glycOMVs). By introducing heterologous carbohydrate biosynthesis pathways, vesicles can be decorated with pathogen-specific polysaccharides such as O-antigens and capsular structures. GlycOMVs mimicking pneumococcal CPS14 induced immune responses comparable to the licensed conjugate vaccine Prevnar13^®^ [77]. Similar platforms have been developed for *Neisseria meningitidis*, *Vibrio cholerae*, and *Acinetobacter baumannii* [78], demonstrating the potential for simplified and cost-effective alternatives to conventional glycoconjugate vaccines.

Scaling production has been facilitated by GMMAs, engineered strains that hyperproduce vesicles through mutations affecting membrane stability (e.g., ΔtolR). GMMA-based vaccines targeting *Shigella*, *Salmonella*, and *Neisseria* have shown promising immunogenicity in both preclinical and clinical studies [79], offering improved yields and manufacturing reproducibility.

Engineered vesicles enriched with protective antigens provide another powerful approach. Vesicles from *Salmonella enterica* engineered to express the *SaoA* protein of *Streptococcus suis* conferred complete protection in murine models [80], while OMVs enriched for type III and VI secretion system proteins from *Burkholderia pseudomallei* elicited strong immunity without the safety concerns associated with live attenuated vaccines [81].

A major frontier is the development of mucosal vaccines. Intranasal delivery of OMVs decorated with the SARS-CoV-2 RBD generated potent systemic IgG and mucosal IgA responses in hamsters, including neutralizing activity against multiple variants [82]. Encapsulation technologies such as biodegradable poly(anhydride) nanoparticles further enhance mucosal stability and provide robust protection via oral or nasal routes [75].

BMVs and synthetic bacterial vesicles (SyBVs) have also shown promise for cancer immunotherapy. Detoxified SyBVs carrying tumor-associated antigens generate strong humoral and cellular responses [83–85], and OMVs engineered to block PD-L1 while delivering tumor epitopes have demonstrated superior antitumor efficacy in preclinical models. Vesicles derived from BCG strains have additionally been shown to induce trained innate immunity [86], offering the possibility of broad-spectrum, heterologous protection.

Together, these innovations demonstrate how BMVs have evolved from pathogen-specific OMV vaccines into highly customizable, safe, and scalable next-generation vaccine systems. Their engineering flexibility, modular antigen-display capabilities, and compatibility with systemic and mucosal delivery routes position BMVs as transformative platforms for vaccines targeting bacterial, viral, and non-infectious diseases.

## Key challenges in BMV vaccine development

The development of BMV-based vaccines still faces several key challenges despite significant technological progress. Safety remains a central concern due to the inherent presence of LPS and other inflammatory components capable of inducing excessive reactogenicity [87, 88]. While genetic engineering approaches, such as lipid A detoxification through targeted mutations have been developed to mitigate this issue, the balance between preserving sufficient adjuvanticity and reducing toxicity remains delicate [87, 89]. Ensuring consistent safety in human populations, especially vulnerable groups such as children or the elderly, continues to be a major regulatory consideration, compounded by reactogenicity testing and batch-to-batch variation challenges [88, 90].

Manufacturing challenges also persist. BMVs are naturally heterogeneous in size, composition, and antigen content, which complicates large-scale production and batch-to-batch standardization [61]. Industrial manufacturing requires reproducible purification processes that maintain vesicle integrity while removing contaminants such as residual DNA, endotoxins, or host cell proteins.

Antigen specificity and variability also present major obstacles. Although BMVs naturally carry a diverse set of bacterial antigens, this repertoire does not always include the most protective epitopes. For pathogens with substantial antigenic diversity, such as *Neisseria meningitidis* or *Shigella*, vesicles derived from a single strain may offer limited cross-protection [76, 91]. While BMVs can be engineered to display conserved or heterologous antigens, such modifications increase technical complexity and raise concerns regarding antigen folding, membrane insertion, structural stability, and correct orientation within the vesicular bilayer [52, 58]. Furthermore, even when antigens are successfully incorporated, their surface accessibility can vary, potentially diminishing the induction of functional, neutralizing antibodies and compromising vaccine efficacy.

Another important challenge is immunodominance and the resulting risk of skewed immune responses. BMVs inherently contain highly abundant bacterial components, such as porins and LPS-associated structures, that can dominate immune recognition and overshadow weaker but essential protective antigens. This may yield strong antibody responses that are not necessarily correlated with protection. Overcoming immunodominance requires advanced antigen-engineering strategies and a precise understanding of correlates of protection. Approaches such as surface display of heterologous antigens using ClyA-based anchoring systems or Plug-and-Display platforms have been utilized to redirect immune recognition toward desired epitopes [61, 92]. However, these methods introduce technical challenges involving antigen folding, membrane insertion, and stable presentation within the vesicle bilayer. Moreover, even when antigens are correctly incorporated, their effective exposure to the immune system may vary, potentially limiting the generation of functional and neutralizing antibody responses [40, 93].

Regulatory pathways for BMV-based vaccines are another emerging challenge. While OMV vaccines against meningococcus have set precedents for licensure [94], new generations of engineered vesicles that carry heterologous or synthetic antigens face more uncertain regulatory scrutiny [92]. Questions regarding standardization of potency assays, acceptable levels of endotoxin activity, and the classification of BMVs as biological products or novel platforms remain partly unresolved [61]. This creates hurdles in translating promising preclinical candidates into licensed vaccines.

From a practical perspective, stability and formulation remain significant hurdles. As complex lipid–protein nanoparticles, BMVs are susceptible to aggregation, structural alteration, and degradation during storage and transport [92, 94]. Ensuring long-term stability, particularly in low-resource settings where cold-chain infrastructure is limited, is critical for global vaccine implementation. Approaches such as lyophilization, microencapsulation, and other advanced formulation technologies can improve stability, but they also introduce additional costs and technical complexities that may hinder large-scale deployment [61]. These challenges are even more pronounced for mucosal vaccine applications, where vesicles must withstand enzymatic degradation, variable pH environments, and mucosal clearance mechanisms before reaching target immune sites.

Finally, host–pathogen interactions and the immunological mechanisms of BMVs are still not fully understood. While it is clear that they stimulate both innate and adaptive immunity [40, 95], the precise pathways, the role of antigen processing and presentation, and the determinants of long-lasting memory remain under investigation [94]. A lack of well-defined correlates of protection complicates the rational design of vesicle vaccines and slows clinical translation. Addressing these knowledge gaps will be essential for refining vesicle-based strategies and for building confidence among regulators and clinicians [92].

## Strategies to overcome challenges in BMV-based vaccines

Looking ahead, several strategies are being actively explored to address the key challenges associated with BMV-based vaccines. A central priority is the fine-tuning of safety. Recent advances in genetic engineering now enable precise modification of lipid A biosynthesis pathways, resulting in “detoxified” vesicles that retain intrinsic adjuvant activity while minimizing systemic reactogenicity [61]. In parallel, synthetic bacterial vesicles generated through mechanical or enzymatic disruption are being evaluated as an alternative means of reducing toxic components without compromising immunogenicity [93]. These approaches aim to achieve an optimal balance between immunostimulation and tolerability, enabling broader and safer application in humans.

In the area of manufacturing and standardization, bioprocess innovations are bringing BMVs closer to industrial scalability. Continuous fermentation systems, improved vesicle-release mutants, and high-yield GMMA strains significantly enhance production capacity [94]. However, downstream processing remains a major bottleneck; methods such as ultracentrifugation, density gradients, filtration, and chromatography must be refined to preserve vesicle structure while minimizing batch-to-batch variability [95]. Complementary to these efforts, standardized analytical assays, measuring vesicle composition, size distribution, antigen density, and residual endotoxin, are under development and will be essential for regulatory acceptance and reproducible large-scale manufacturing [92].

To overcome the limitations of antigen coverage and strain specificity, modular engineering strategies are increasingly being adopted. Fusing heterologous proteins or polysaccharides to vesicle-associated scaffolds enables the presentation of conserved epitopes across multiple strains or pathogens [56]. Glycoengineered OMVs carrying capsular or O-antigen polysaccharides offer a promising, cost-effective alternative to traditional glycoconjugate vaccines [40]. Furthermore, plug-and-play antigen exchange platforms facilitate rapid adaptation to emerging infectious threats, paralleling the responsiveness demonstrated by mRNA technologies during the COVID-19 pandemic [61].

Progress is also being made toward addressing immunodominance. Selective deletion or downregulation of highly abundant but non-protective antigens allows immune responses to be redirected toward desired targets [92]. Advances in systems immunology and high-throughput epitope mapping are improving the definition of protective correlates, thereby enabling more rational vesicle design [56]. When combined with optimized adjuvant formulations or targeted delivery routes, these efforts can help ensure that BMV-based vaccines elicit not only robust but also protective immunity.

Formulation and stability challenges are being tackled through several innovative delivery systems. Lyophilized vesicle powders, biodegradable nanoparticle encapsulation, and specialized mucosal formulations all show promise for enhancing stability and enabling needle-free administration via oral or intranasal routes [61, 93]. Such approaches could vastly expand the applicability of BMV-based vaccines, particularly in low-resource settings where cold-chain infrastructure or injection-based delivery pose barriers.

Finally, regulatory clarity is expected to improve as more candidates progress through clinical trials. The successful licensure of meningococcal OMV vaccines and the ongoing development of Shigella GMMA vaccines offer important precedents for next-generation vesicle platforms [61, 94]. As standardized potency assays are validated and mechanistic understanding of vesicle-induced immunity deepens, regulatory agencies will be better positioned to evaluate these vaccines and support their clinical translation.

## Future perspectives

Future research on BMVs as vaccine platforms should focus on deepening the mechanistic understanding of the immune responses they generate. Although BMVs are well-recognized for their strong induction of both innate and adaptive immunity, the correlates of protection and the mechanisms governing long-term immunological memory remain insufficiently defined. High-throughput immunoprofiling, single-cell transcriptomics, and systems immunology approaches will be valuable for mapping the immune pathways activated by BMVs. Such mechanistic insights will support rational engineering strategies to enrich protective antigens while minimizing non-protective or reactogenic components.

Advancing genetic engineering capabilities will also be essential. CRISPR-based genome editing, synthetic biology tools, and modular plug-and-play scaffolds represent powerful approaches to fine-tune vesicle composition [92]. Systematic deletion of immunodominant but non-protective proteins or the incorporation of conserved epitopes from heterologous pathogens can further optimize vesicle safety and efficacy. Expansion of glycoengineering platforms, particularly for polysaccharide presentation, offers opportunities for developing cost-effective alternatives to traditional glycoconjugates [40].

Equally important will be the establishment of standardized characterization and quality-control assays. Widely accepted metrics for vesicle size distribution, antigen density, endotoxin activity, and immune potency are needed to strengthen reproducibility across laboratories and facilitate regulatory approval. Shared databases cataloging BMV composition, immune profiles, and clinical outcomes would further enhance transparency and accelerate field-wide progress.

Given the interdisciplinary nature of BMV science, integration across microbiology, immunology, synthetic biology, nanotechnology, and translational medicine will be critical. Collaborative research networks and open-access consortia can help resolve complex engineering challenges, improve manufacturing scalability, and streamline preclinical-to-clinical translation.

In summary, the future of BMV-based vaccines will be shaped by synergistic advances in genetic engineering, bioprocess optimization, modular antigen design, formulation science, and regulatory harmonization. As these elements converge, BMVs may transition from a specialized research tool into a broadly applicable vaccine platform with substantial impact across bacterial, viral, and non-infectious disease targets. With sustained innovation, BMVs have the potential to stand alongside mRNA and viral-vector technologies as one of the defining vaccine platforms of the 21st century.

## Conclusion

BMVs have evolved into highly versatile, next-generation vaccine platforms, far surpassing first-generation meningococcal OMV vaccines. Advances in genetic detoxification, including targeted lipid A modifications (msbB, htrB, lpxL), have successfully reduced endotoxin-mediated reactogenicity while preserving intrinsic adjuvant properties. Glycoengineering strategies now allow BMVs to display capsular polysaccharides and O-antigens, offering low-cost alternatives to classical glycoconjugate vaccines.

Modular antigen display systems, such as SpyTag/SpyCatcher, ClyA fusions, and Hbp scaffolds, enable rapid incorporation of heterologous proteins, viral antigens, or tumor-associated epitopes in their native conformations, transforming BMVs into true “plug-and-play” platforms capable of fast responses to emerging pathogens. High-yield GMMA strains, continuous fermentation, and advanced downstream purification technologies have improved production scalability, consistency, and regulatory readiness.

Innovations in formulation, including lyophilization, nanoparticle encapsulation, and mucosal delivery systems, are expanding BMVs beyond traditional injectable vaccines, toward needle-free, thermostable formats suitable for global deployment. With their intrinsic adjuvanticity, broad antigenic presentation, and growing industrial feasibility, BMVs are poised to join mRNA and viral vector vaccines as a defining technology of 21st-century vaccinology.

## Data Availability

No datasets were generated or analysed during the current study.
